# Gap junction intercellular communications regulates activation of SARM1 and protects against axonal degeneration

**DOI:** 10.1038/s41419-025-07342-4

**Published:** 2025-01-14

**Authors:** Wen Jie Zhu, Jun Liu, Wan Hua Li, Zhi Ying Zhao, Chongquan Huang, Jian Yuan Yang, Hon Cheung Lee, Yong Juan Zhao

**Affiliations:** 1 State Key Laboratory of Chemical Oncogenomics, Key Laboratory of Chemical Genomics, Peking University Shenzhen Graduate School, Shenzhen, 518055 China; 2 Ciechanover Institute of Precision and Regenerative Medicine, School of Life and Health Sciences, School of Medicine, The Chinese University of Hong Kong, Shenzhen, 518172 China

**Keywords:** Cell biology, Molecular biology

## Abstract

Sterile alpha and Toll/interleukin-1 receptor motif containing 1 (SARM1), a nicotinamide adenine dinucleotide (NAD)-utilizing enzyme, mediates axon degeneration (AxD) in various neurodegenerative diseases. It is activated by nicotinamide mononucleotide (NMN) to produce a calcium messenger, cyclic ADP-ribose (cADPR). This activity is blocked by elevated NAD level. Here, we verified this metabolic regulation in somatic HEK-293T cells by overexpressing NMN-adenyltransferase to elevate cellular NAD, which resulted not only in inhibition of their own SARM1 from producing cADPR but, surprisingly, also in the 5–10 neighboring wildtype cells in mixed cultures via connexin (Cx)-43. Direct visualization of gap junction intercellular communication (GJIC) was achieved by incubating cells with a permeant probe, PC11, which is converted by SARM1 into PAD11, a fluorescent NAD analog capable of traversing GJs. Extending the findings to dorsal root ganglion neurons, we further showed that CZ-48, a permeant NMN analog, or axotomy, activated SARM1 and the produced PAD11 was transferred to contacting axons via GJIC. The gap junction involved was identified as Cx36 instead. This neuronal GJIC was demonstrated to be functional, enabling healthy neurons to protect adjacent axotomized axons from degeneration. Inhibition of GJIC in mice by AAV-PHP.eB-mediated knockdown of Cx36 in brain induced neuroinflammation, which in turn activated SARM1 and resulted in axon degeneration as well as behavioral deficits. Our results demonstrate a novel intercellular regulation mechanism of SARM1 and reveal a protective role of healthy tissue against AxD induced by injury or neuroinflammation.

## Introduction

Axon degeneration (AxD) is a critical process underlying various neurological diseases, including peripheral neuropathies [1], traumatic brain injury [2]. amyotrophic lateral sclerosis [3], Parkinson’s disease [4], and glaucoma [5]. Understanding the mechanisms underlying AxD is crucial for the development of therapeutic strategies to halt or delay disease progression. The discovery and characterization of Wallerian Degeneration Slow (Wld^s^) spontaneous mutant mice have shed light on the pivotal role of NAD metabolism in initiating and mediating AxD [6, 7]. These mice ectopically express NMNAT1, a nuclear NAD synthase, in cytosol, which blocks injury-induced AxD.

Sterile Alpha and TIR Motif-Containing 1 (SARM1) has emerged as a central mediator of AxD. Genetic screening studies demonstrated that *Drosophila* carrying the mutations in dSarm, the homolog of SARM1, exhibited a significant delay in AxD [8]. The beneficial effects of SARM1 knockout have been confirmed in different rodent disease models, such as chemotherapy- or diabetes-induced peripheral neuropathy [9–11], traumatic brain injury [12], glaucoma [13], amyotrophic lateral sclerosis [14], and Charcot-Maries-Tooth disease [15]. SARM1 is primarily expressed in neurons and comprises three domains: an Armadillo repeat motif (ARM) domain responsible for autoinhibition [16, 17], a sterile alpha motif (SAM) domain responsible for octamerization [18], and a C-terminal Toll/interleukin-1 receptor (TIR) domain possessing NADase activity [19]. Additionally, SARM1 possesses an N-terminal 27-aa mitochondrial localization signal, targeting it to the outer mitochondrial membrane [20], although some reports also showed a distinctly non-mitochondrial location [8, 16].

Substantial progress has been made in understanding the regulatory mechanisms of SARM1 in recent years. Our studies have revealed that nicotinamide mononucleotide (NMN), along with its cell permeant analog, CZ-48, activates SARM1 by relieving the autoinhibition imposed by the ARM domain [21]. Structural investigations have provided critical evidence that NAD functions as an endogenous inhibitor, maintaining SARM1 in an autoinhibitory conformation [22, 23]. Both NMN and NAD bind to an allosteric site within the ARM domain but exert opposite regulatory effects on SARM1 [22, 24–26]. The balance between NMN and NAD determines SARM1 activation and subsequent cellular events such as AxD [24]. These findings have established a working model for AxD, whereby injury or damage to axons triggers the rapid loss of NMNAT2, an enzyme responsible for NAD synthesis from NMN and ATP, leading to NMN accumulation [27, 28] and NAD decrease, which subsequently activates SARM1, further depleting NAD and ultimately causing axon fragmentation [29].

Given the critical role of SARM1 in AxD-related neurodegenerative disease and the advancements in understanding its regulatory mechanisms, it is crucial to explore novel avenues of SARM1 modulation. This manuscript aims to investigate the potential influence of inter-neuronal gap junction (GJ) communication on SARM1 regulation. Gap junction intercellular communication (GJIC), facilitating direct cell-to-cell exchange of ions and metabolites, has been extensively studied in both somatic and neuronal systems [30]. Neuronal GJs play essential roles in neurodevelopment and disease processes [31, 32]. During injury, GJs are unregulated and can have either “pro-death” or “pro-survival” effects depending on the specific molecules they transport [32]. However, the intercellular communication of NAD or related metabolites through GJs and its impact on SARM1 activity and AxD have not been explored. Here we show a novel intercellular regulation mechanism of SARM1 and reveal a protective role of healthy tissue against AxD upon injury or neuroinflammation.

## Results

### NMNAT1-overexpressing cells inhibit SARM1 activation in neighboring cells through gap junction intercellular communication

**Fig. 1:**
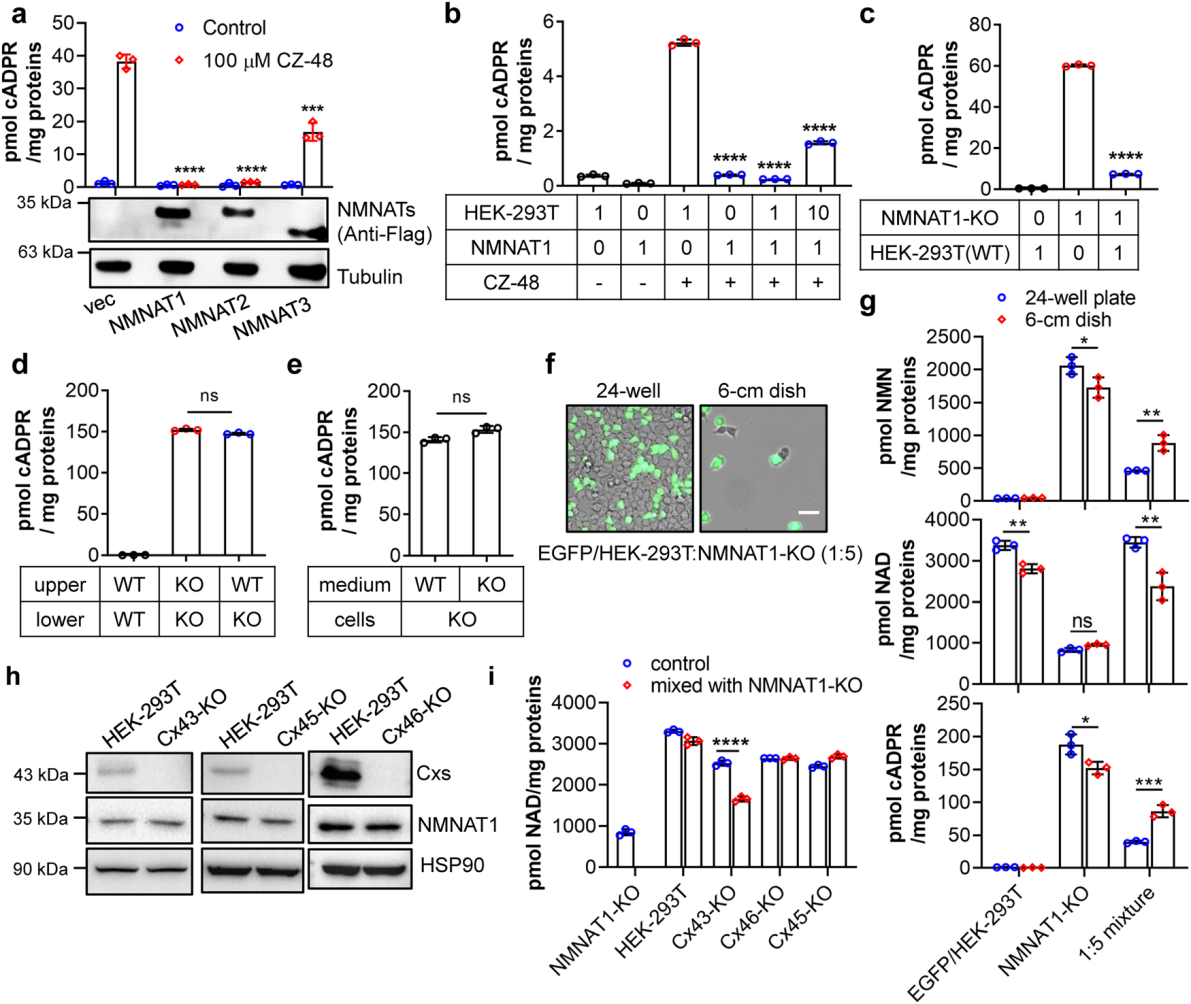
NMNAT1-positive cells inhibit SARM1 activation in neighboring cells through gap junction intercellular communication. **a** HEK-293T cells were transfected with expression vectors encoding Flag-tagged NMNATs or an empty vector control (Vec). The cells were subsequently treated with 100 μM CZ-48 or PBS and incubated for 24 h before being lysed. Levels of cADPR and proteins were measured using a cycling assay and Western blot, respectively. **b** Wildtype and NMNAT1-overexpressing HEK-293T cells were mixed in the indicated ratio and treated with or without 100 μM CZ-48 for 24 h. cADPR contents were measured using the cycling assay. **c** Wildtype and NMNAT1-knockout HEK-293T cells were mixed in the indicated ratio and cultured for 24 h, followed by cADPR measurement. **d** Wildtype and NMNAT1-knockout HEK-293T cells were co-cultured in a Boyden Chamber, with one cell line in the upper chamber and another in the lower chamber, for 24 h. cADPR levels were measured in the cells in the lower chamber. **e** NMNAT1-KO HEK-293T cells were cultured with the conditioned medium from wildtype or NMNAT1-knockout cells for 24 h, followed by cADPR measurement. **f**, **g** HEK-293T cells expressing EGFP were co-cultured with NMNAT1-knockout HEK-293T cells in a 1:5 ratio in a 24-well plate or 6-cm dish. After 24 h, fluorescent or bright-field images were captured under a fluorescent microscope (**f**), and the levels of NMN, NAD, and cADPR (**g**) were measured by the cycling assay. Scale bar: 20 μm. **h** Western blot of Cx43, Cx45 and Cx46 proteins in wildtype or knockout cells generated by the CRISPR/cas9 technique. **i** NAD levels were measured in connexin-knockout cells or a 1:1 mixture of connexin-knockout and NMNAT1-knockout cells. All experiments were repeated at least three times, and data are presented as means ± SDs (n = 3). *P* values (ns, *P* > 0.05; ***P < 0.001; ****P < 0.0001) were determined by Student’s *t* test. One-way ANOVA tests were performed in **a**–**d** before *t*-test.

We first verified the metabolic regulation of SARM1 is operational in cells. SARM1 activity was monitored by measuring its enzymatic product, cADPR, following activation by CZ-48 [21]. Overexpressing the nuclear NMNAT1 [33] or Golgi-localized NMNAT2 [33] inhibited SARM1 activation (Fig. 1a and Supplementary Fig. S1a), probably due to their NAD synthase activity, which prevented a significant decline in NAD upon CZ-48 treatment (Supplementary Fig. S1b). The mitochondrial NMNAT3 [33] was less effective (Fig. 1a and Supplementary Fig. S1a, b). To determine whether the NAD synthesis activity of the NMNAT1 was responsible for the inhibition, we mutated several residues within or surrounding the catalytic pocket of NMNAT1 (Supplementary Fig. S1c) to glycine. These mutants exhibited different activities in inhibiting the CZ-48-induced cADPR production in HEK-293T cells (Supplementary Fig. S1d, red dots), which was negatively correlated with their NAD synthetase activities (Supplementary Fig. S1d, blue dots), indicating that NAD synthesis activity was indeed responsible. This is consistent with the report that NAD binds to the ARM domain and mediates self-inhibition of SARM1 [22]. Conversely, knocking out NMNAT1 elevated cellular NMN and activated SARM1, which was reversed by re-expression of NMNATs (Supplementary Fig. S1e), verifying that the metabolic regulation of SARM1 by the cellular levels of NMN and NAD [21, 22, 24] is indeed operational in cells. The less effectiveness of NMNAT3 may be due to the low level of mitochondrial transporter of NAD, SLC25A51 in HEK-293T [34], hampering the transfer of the mitochondrial NAD to the cytosol.

In the above experiments, even with only approximately 50% of cells showing positive NMNATs staining (Supplementary Fig. S1a), complete inhibition of cADPR production in the entire culture was observed (Fig. 1a), suggesting possible intercellular transfer of inhibition. To quantitatively document this effect, we mixed different ratios of wildtype HEK-293T cells expressing low levels of NMNATs with NMNAT1-overexpressing stable cell lines. Treatment with CZ-48 induced cADPR production in wildtype HEK-293T cells but not in NMNAT1-overexpressing cells (Fig. 1b, left 4 columns). Notably, the 1:1 mixture displayed no cADPR production, and 10:1 mixture exhibited approximately one-third of the cADPR amount compared to the HEK-293T group (Fig. 1b, right 2 columns). These results demonstrate that NMNAT1 not only inhibits CZ-48-induced SARM1 activation and subsequent cADPR production in its own cells but also in neighboring cells in the culture.

We further investigated this phenomenon using a different system, by mixing NMNAT1-KO HEK-293T cells with wildtype HEK-293T cells. The NMNAT1-KO cells displayed remarkably high levels of cADPR, shown also in Supplementary Fig. S1e, much higher than the wildtype HEK-293T cells. Upon mixing the two cell types in a 1:1 ratio, only minimal cADPR production was observed (Fig. 1c). This finding suggests that the endogenous NMNAT1 in wildtype HEK-293T cells can inhibit NMN-induced SARM1 activation in NMNAT1-KO cells.

Since cells communicate through various mechanisms, we sought to determine whether the above phenomenon requires cell-cell contact. We conducted additional experiments to address this question. Firstly, we seeded the two cell lines separately in two chambers of a Boyden Chamber and measured the cellular cADPR levels in the lower chamber. In this configuration, the wildtype HEK-293T cells did not inhibit cADPR production in NMNAT1-KO cells (Fig. 1d). Secondly, we cultured NMNAT1-KO cells with the conditioned medium of the two cell lines. Interestingly, the conditional medium from wildtype HEK-293T cells did not inhibit cADPR production in NMNAT1-KO cells (Fig. 1e). These results strongly suggest that direct cell-cell contact is required for the intercellular regulation of SARM1 activation observed in our experiments.

To further explore the role of cell-cell contact, we co-seeded an equal number of the 1:5 mixed HEK-293T cells expressing EGFP and NMNAT1-KO cells in wells with different surface areas, specifically a 24-well plate and a 6-cm dish, resulting in approximately a 10-fold difference in cell density (Fig. 1f). In the 24-well plate, where all cells formed contacts with other cells, the healthy HEK-293T cells (EGFP positive) efficiently alleviated NMNAT1-KO-induced NMN accumulation and NAD depletion, subsequently inhibited cADPR production in the mixed population (Fig. 1g, blue dots). These findings are consistent with our previous observations (Fig. 1a–e). In contrast, in the 6-cm dish, where around half of the population grew as single cells without cell-cell contacts, the mixture showed similar trends in nucleotide levels, but to a significantly lesser extent (Fig. 1g, red dots). This suggests that the inhibitory effect quantitatively depends on the presence of cell contacts.

To investigate the potential involvement of gap junctions in mediating the observed inhibitory effect, we performed microarray analysis of HEK-293T cells, which revealed relatively high transcriptional levels of three connexins, including connexin 43 (Cx43), connexin 45 (Cx45) and connexin 46 (Cx46). We subsequently employed CRISPR/cas9 technology to knockout these genes in HEK-293T cells. The knockout cells were validated through Western blot analysis using specific antibodies against connexins (Fig. 1h) and then mixed with NMNAT1-KO cells. Interestingly, Cx43-KO cells exhibited a significantly reduced ability to replenish the NAD store in NMNAT1-KO cells compared to wildtype HEK-293T cells, while knockout of the other two connexins did not affect the inhibitory effect (Fig. 1i). These data suggest that the intercellular regulation of SARM1 activation among HEK-293T cells occurs through gap junctions, predominantly by Cx43.

### Visualization of intercellular communication of nucleotides via gap junctions

**Fig. 2:**
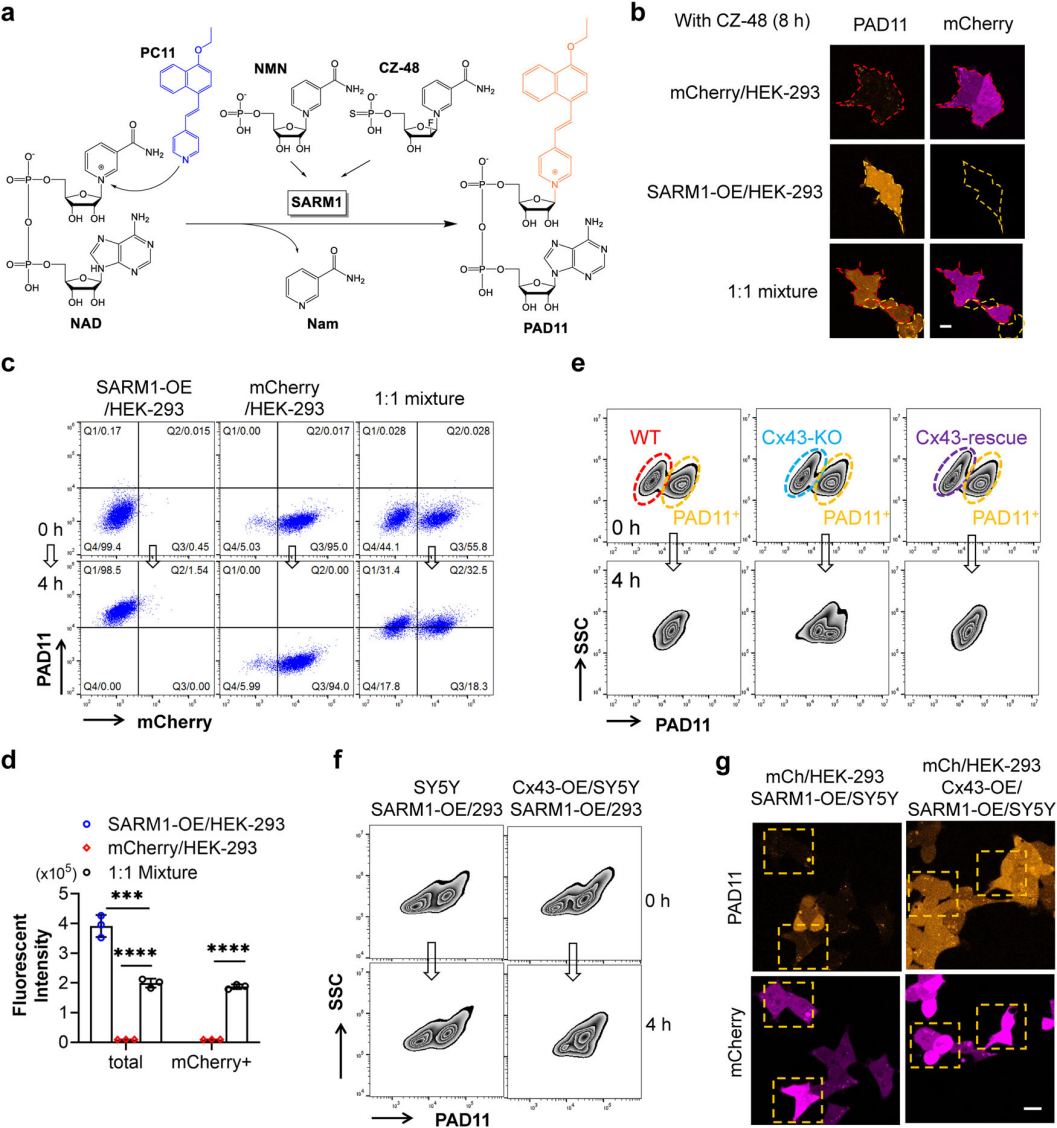
Visualization of intercellular communication of nucleotides mediated by gap junction. **a** The base-exchange reaction catalyzed by SARM1. PC11, the base, and CZ-48, the activator, are artificially designed and synthesized, while NAD, NMN, and Nam are endogenous nucleotides. **b** HEK-293 cells expressing mCherry (labeled with red edges) and cells overexpressing SARM1 (orange edges) were cultured separately or as a 1:1 mixture. The cells were treated with 100 μM CZ-48 and 12.5 μM PC11 for 8 h and the images were captured under a confocal microscope. Cell edges were marked according to the bright-field images. Scale bar: 10 μm. **c** The same cells as in **b** were treated with 12.5 μM PC11, with or without 100 μM CZ-48, for 4 h and the fluorescence of mCherry and PAD11 was analyzed by flow cytometry. **d** Fluorescence intensity of the Q1 + Q2 areas in the mixed cell population from **c**. **e** NMNAT1-knockout HEK-293T cells, pre-treated with 12.5 μM PC11 for 16 h, were mixed with wildtype HEK-293T cells, or Cx43-knockout HEK-293T cells, or Cx43-rescued HEK-293T cells, and co-cultured for 4 h. The fluorescence of PAD11 were analyzed by flow cytometry. SSC: side scatter, indicating granularity of the cell population. Red circle: wildtype HEK-293T cell population; blue circle: Cx43-knockout cells; purple circle: Cx43-rescued cells; orange circle: NMNAT1-knockout cells. **f** SARM1-OE HEK-293 cells, pre-treated with 12.5 μM PC11 for 16 h, were mixed with wildtype SH-SY5Y cells, or Cx43-OE SH-SY5Y cells in a 1:1 ratio, and co-cultured for 4 h. The PAD11 fluorescence was analyzed by flow cytometry. **g** HEK-293 cells expressing mCherry were co-cultured with SARM1-OE SH-SY5Y cells, or SARM1/Cx43-OE SH-SY5Y cells in a 1:1 ratio. The cells were treated with 100 μM CZ-48 and 12.5 μM PC11 for 8 h and the images were captured under a confocal microscope. Wildtype HEK-293 cells (mCherry positive) contacting with SARM1-OE cells were highlighted with orange squares. Scale bar: 10 μm. All experiments were repeated at least three times, and data are presented as means ± SDs (n = 3). Statistical significance was determined using Student’s *t* test in **d** (***P < 0.001; ****P < 0.0001); one-way ANOVA test in the “total” group was performed before *t*-test.

Previously, we have developed a series of fluorescent probes that are not only highly sensitive to measure the activity of SARM1 in vitro but also capable of imaging the activity in live cells induced by CZ-48, vincristine, or axotomy [35, 36]. Here, we employed PC11 (Probe 1a in literature [36]) to visualize intercellular nucleotide communication as well. PC11 undergoes a catalytic base exchange with the nicotinamide (Nam) group in the NAD molecule. The reaction is selectively catalyzed by NMN- or CZ-48-activated SARM1, resulting in the formation of a fluorescent analog of NAD called PAD11 (Fig. 2a). It is a highly charged molecule and is thus cell impermeant and can only be transferred via GJs to other cells. By monitoring the fluorescence of PAD11, we can visualize its intracellular transfer, and in inference, that of nucleotides with similar size and charges, such as NAD, NMN or Nam.

HEK-293 cells overexpressing SARM1, treated with 100 μM CZ-48 and 12.5 μM PC11 for 8 h, exhibited a bright orange fluorescence due to the accumulation of PAD11 (Fig. 2b, second panel). In contrast, wildtype HEK-293 cells, expressing low levels of endogenous SARM1 [21] and exogenous mCherry as a marker, did not display observable PAD11 signals (Fig. 2b, first panel). Interestingly, when these two stable cell lines were mixed in a 1:1 ratio, all the cells within the same cluster, comprising both mCherry-positive (wildtype) and mCherry-negative (SARM1-OE) HEK-293 cells, exhibited similar intensities of PAD11 signals (Fig. 2b, third panel). We observed three distinct types of cell clusters in the same field of the mixture (Supplementary Fig. S2a), where the mCherry-positive cluster did not show PAD11 signals (red square), the mCherry-negative cluster exhibited PAD11 fluorescence (orange square), and the pink squared cluster consisted of mCherry-positive cells acquiring PAD11 through contact with SARM1-OE cells. These findings confirmed that nucleotides can indeed travel among cells.

Quantification of the same experiments using flow cytometric analyses is shown in Fig. 2c. Activation by CZ-48 for 4 h of SARM-OE greatly increased PAD11 fluorescence (Fig. 2c, left panel). Very low PAD11 fluorescence was seen in wildtype (Fig. 2c, middle panel, mCherry positive) due to low expression of endogenous SARM1. When the two cell types were mixed one to one, PAD11 was seen in all cells (Fig. 2c, right panel), even though the PAD11 fluorescence was lower than in the culture of SARM1-OE alone (Fig. 2c, d). This suggested that the PAD11 molecules produced by SARM1-OE cells were diluted by wildtype HEK-293 cells and confirmed the transfer of nucleotides between cells.

To rule out the possibility of SARM1 transfer with mitochondrion through tunneling nanotubes [37], we stained the mixed cells with a SARM1 antibody. The results showed exclusive presence of SARM1 signals in mCherry-negative cells (Supplementary Fig. S2b).

To further validate the involvement of Cx43 in intercellular communication, we performed co-culture experiments using NMNAT1-KO HEK-293T cells and either Cx43-KO cells or wildtype cells as control, in the presence of PC11. In the control co-culture, PAD11 fluorescence levels became similar between the two cell populations after 4 h (Fig. 2e, left panel). However, this equalization process was significantly impaired in the Cx43-KO co-culture (Fig. 2e, middle panel), and the normal communication was restored upon re-expression of Cx43 (Fig. 2e, right panel). We quantified the communication between the two cell populations by measuring the reduction in the fluorescence intensity difference, as shown in Supplementary Fig. S2f, g. The expression of Cx43 in the cells was confirmed by Western blot (Supplementary Fig. S2c). These findings provide additional evidence supporting the role of Cx43 in facilitating nucleotides trafficking among cells.

Interestingly, we observed that PAD11 did not transfer between two different cell types, HEK-293 and SH-SY5Y (Fig. 2f, g, left panels). We reasoned that this could be attributed to the differential expression of connexin isoforms. According to the RNAseq data, HEK-293 cells predominantly express Cx43; while SH-SY5Y cells mainly express Cx45. Consistent with this, overexpression of Cx43 in SH-SY5Y cells facilitated intercellular communication of PAD11 between the two different cell types (Fig. 2f, g, right panels). The communication was confirmed by quantifying the reduction in the fluorescence intensity difference between two cell populations, as shown in Supplementary Fig. S2h. And the expression of Cx43 in the cells was verified by Western blot (Supplementary Fig. S2d, e). These findings suggest that the expression of the same connexin isoforms expressing in adjacent cells mediates intercellular nucleotide exchange.

### Visualization of inter-neuronal gap junction communication of nucleotides

**Fig. 3:**
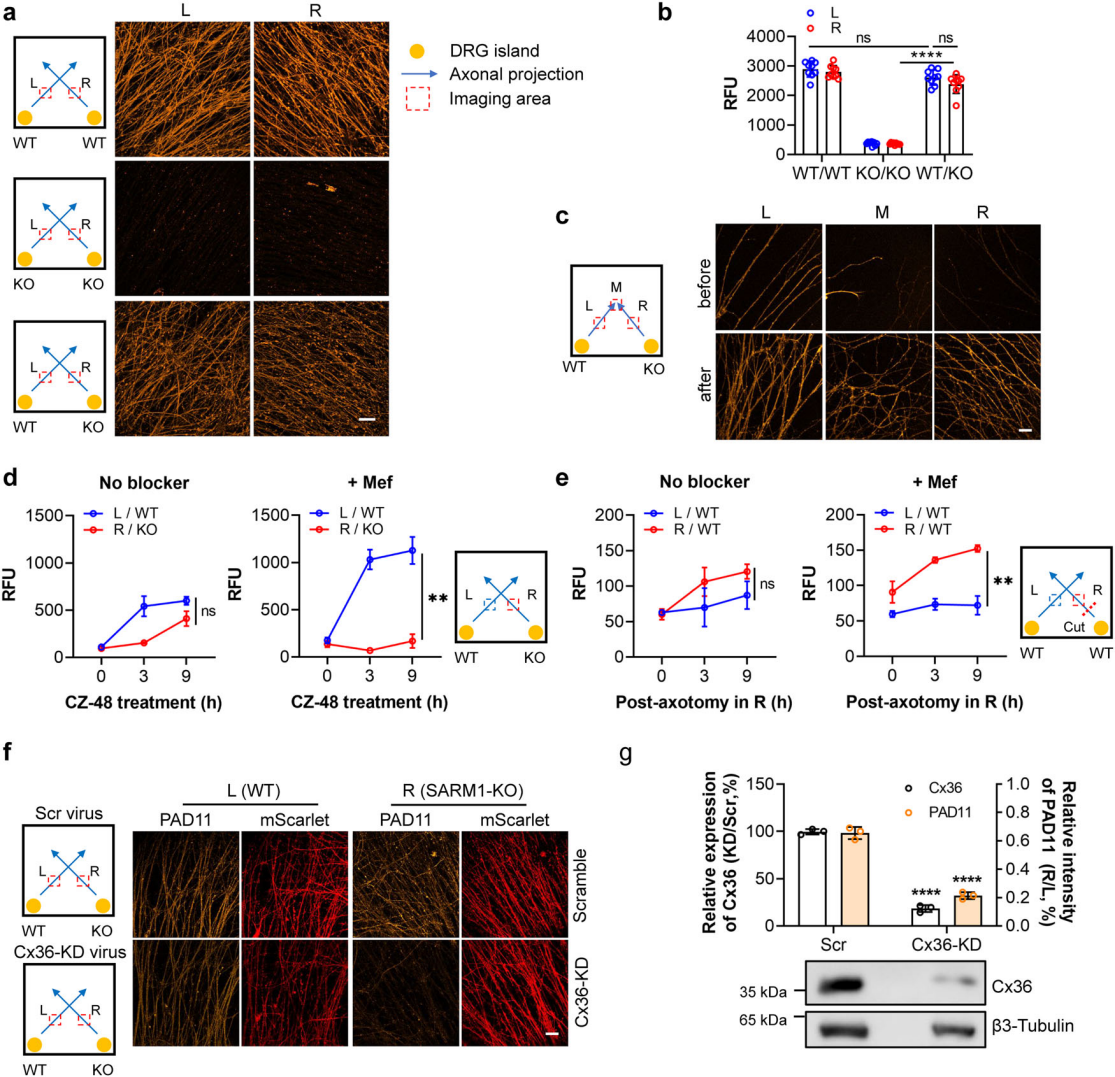
Visualization of inter-neuronal communication of nucleotides mediated by gap junctions. **a** Wildtype or SARM1-knockout DRG neurons were seeded in two corners of a well of an 8-well Chambered Coverglass as two small islands (represented by orange dots in the illustration) and cultured until the outgrowing axons contacted each other. The culture was treated with 25 μM PC11, with or without 200 μM CZ-48, for 16 h. Fluorescence signals in the axons within the red-dotted areas were captured using a confocal microscope. “L” and “R” represent the axons projected from the left and right islands, respectively. Scale bar: 10 μm. **b** Quantification of fluorescence intensity in the images from **a**. **c** Similar experiments to the third-row co-culture in **a** were conducted, except that the images were captured before or after the axons contacted. Scale bar: 10 μm. **d**, **e** Wildtype DRG neurons were seeded as two islands. When the axons projected from these islands made contact, the cultures were pretreated with 5 μM mefloquine (right charts) or vehicle as a control (left charts) for 2 h, followed by the addition of 200 μM CZ-48 (**d**) or axotomy (**e**), together with 25 μM PC11. Fluorescence of PAD11 within the squared areas was monitored at the indicated time points, and fluorescence intensity was quantified. **f** Wildtype and SARM1-knockout DRG neurons were seeded as a third-row co-culture in **a** and infected with AAV-PHP.eB encoding Cx36-targeting shRNA or a scramble shRNA, indicated by mScarlet expression. The culture was treated with 25 μM PC11 and 200 μM CZ-48, for 6 h, and fluorescence of PAD11 and mScarlet was captured under a confocal microscope. Scale bar: 10 μm. **g** Endogenous expression level of Cx36 in the DRG neurons in **f** was measured by Western blot. Quantification of the PAD11 fluorescence intensity in **f**. Signals from the right side were normalized to those from the co-cultured left axons. All experiments were repeated at least three times, and data are presented as means ± SDs (n = 3). Statistical significance was determined by Student’s *t* test in **b**, following two-way ANOVA test; Student’s *t* test in **d**, **e**, and **g** (ns no significance; **P < 0.01; ***P < 0.001; ****P < 0.0001).

SARM1 is a crucial NAD-utilizing enzyme primarily expressed in neurons, playing a key role in axon degeneration [8, 19]. Results and characterization of its intercellular regulation in the somatic HEK-293 cells provide the needed information to extend the study to neurons. To address this, we established co-cultures of dissociated dorsal root ganglion (DRG) neurons isolated from wildtype or SARM1-knockout mice. Two cell aggregates, representing WT or KO neurons, were positioned in separate corners of the well (Fig. 3a, orange circles in the illustration) and allowed to develop axonal projections until they made contacts (blue arrows in the illustration). Subsequently, the cultures were treated with PC11 in the presence or absence of the activator CZ-48, and after 16 h, the areas prior to contacts zone (red dotted square in the illustration) were imaged using a confocal microscope. Consistent with our previous observation [35, 36], PC11 stained the axons derived from wildtype DRG neurons with CZ-48 treatment, but not those from SARM1-knockout neurons (Fig. 3a, first two rows) or without CZ-48 treatment. However, when SARM1-knockout neurons were co-cultured with wildtype neurons and axonal contacts were established, the SARM1-KO axons also exhibited the orange fluorescence indicative of PAD11 (Fig. 3a, 3^rd^ row). Quantification analysis clearly demonstrated that SARM1-knockout axons in contact with wildtype axons displays a similar amount of PAD11 fluorescence compared to wildtype axons, in contrast to the lack of staining when in contact with knockout axons (Fig. 3b).

To confirm that this communication is dependent on neuronal contacts, we performed time-series imaging. Prior to contacting with wildtype axons, the SARM1-knockout axons exhibited no PAD11 signals (Fig. 3c, upper panel). However, once contacts were formed, they displayed similar brightness of PAD11 signals to the neighboring wildtype axons (Fig. 3c, lower panel).

Neurons express several types of connexins, including Cx36, Cx50 and Cx45 [38], while Cx36 is the major form [39]. In the co-culture system of wildtype and SARM1-KO neurons, the addition of the Cx36 inhibitor mefloquine (Mef) significantly delayed the communication of PAD11, which was synthesized in wildtype axons induced by CZ-48 (Fig. 3d) or axotomy (Fig. 3e).

To further support the role of Cx36, we constructed an AAV-PHP.eB virus carrying an mScarlet expression cassette as a reporting gene, along with the shRNA targeting *Cx36* gene (KD), or a scramble sequence as a control. Neurons infected with Cx36-KD virus exhibited significantly lower PAD11 signals in SARM1-KO cells compared to those infected with Scramble viruses (Fig. 3f, g). Western blot analysis confirmed efficient knockdown of the Cx36 gene in the Cx36-KD group (Fig. 3g, lower panel), confirming that Cx36 mediates GJIC of nucleotides in DRG axons.

### Inter-neuronal communication regulates axon degeneration

**Fig. 4:**
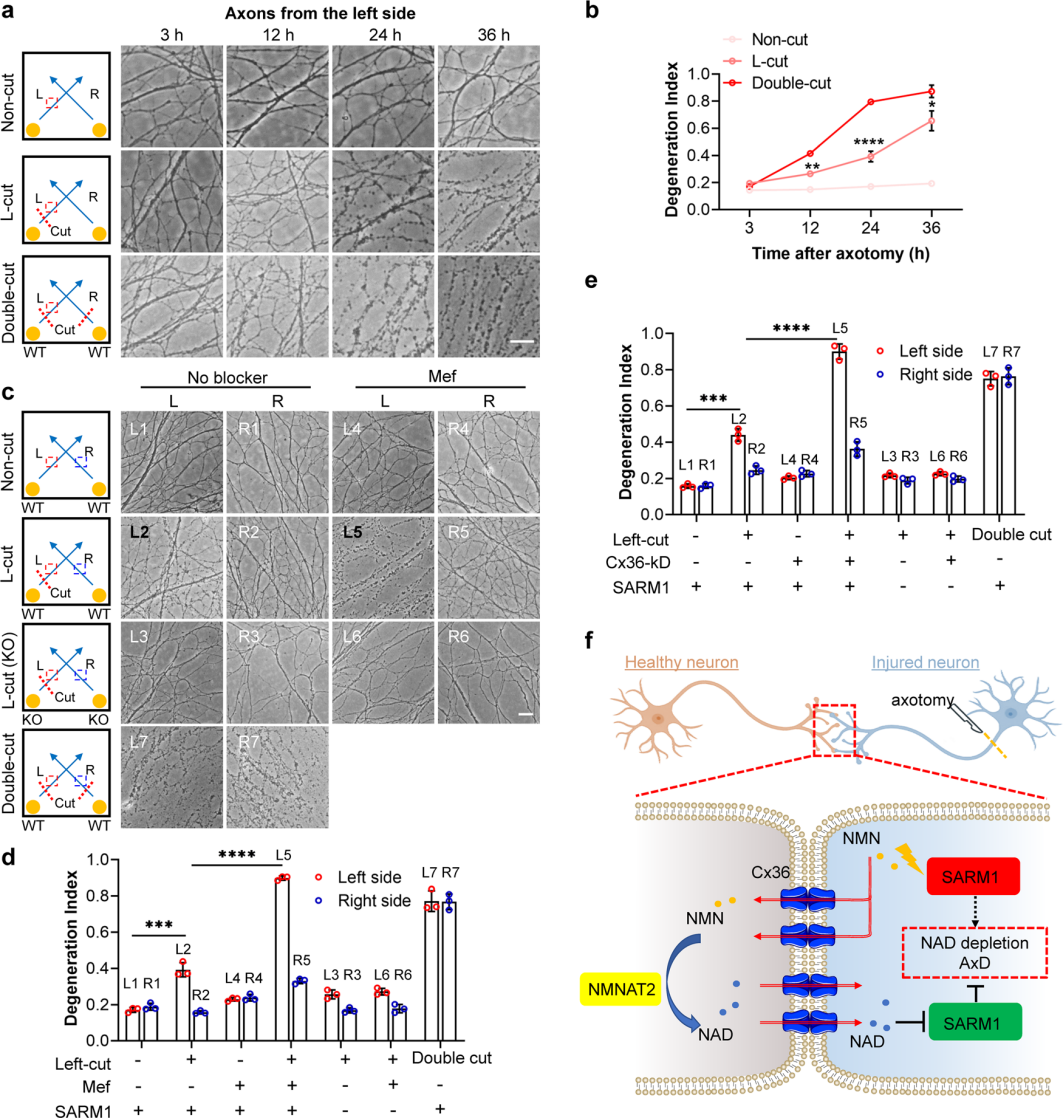
Gap junction-mediated inter-neuronal communication regulates axon degeneration. **a** Wildtype DRG neurons were cultured as two separate islands, allowing axonal contact to form. Axotomy was then performed at the indicated sites (denoted by dashed lines), and bright-field microscope images were captured at specified times. Scale bar: 10 μm. **b** The degeneration index of axons from the left island was quantified using ImageJ and plotted over time. **c** Wildtype or SARM1-KO DRG neurons were seeded. Following the axonal contacts, the cultures were pretreated with 5 μM mefloquine, and the axotomy were conducted. 24 h later, images were taken. **d** Degeneration indices of the axons from **c**, were calculated and plotted. **e** Experiments were performed as in **c**, but with infection with AAV-PHP.eB encoding Cx36-specific shRNA or scramble shRNA instead of mef treatment. Images were shown in Supplementary Fig. S3c. Degeneration indices were calculated and plotted. All experiments were repeated at least three times, and data are presented as means ± SDs (n = 3). Statistical significance was determined using Student’s *t* test following two-way ANOVA in **b**; using Student’s *t* test with Bonferroni correction (*P* < 0.0024, 0.05/21) in **e** and **d** (ns no significance; ***P < 0.001; ****P < 0.0001). **f** A proposed model illustrating the intercellular regulation of SARM1 activation was shown.

The possibility that healthy axons can exert protective effects on adjacent injured ones, through the intercellular transfer of NAD and its metabolites, was investigated. Wildtype DRG neurons were seeded as two separated clusters and cultured until their axons made contacts. The axons on the left side were cut, while those on the right side remained uninjured (Fig. 4a, 2^nd^ row). Additionally, cultures without any cut (Non-cut) and with double cuts (Double-cut) served as negative and positive controls, respectively (Fig. 4a, 1^st^ and 3^rd^ rows). After culturing for various time periods, images were captured and degeneration indices were calculated. The progression of axonal degeneration, as shown in Fig. 4a, b, clearly demonstrated that the presence of contacting healthy neurons (in L-cut) delayed the degeneration process compared to uniformly injured ones, while the healthy axons (in L-cut) at the right side remained intact, showing little negative impact from the injured ones (Supplementary Fig. S3a, b).

We then repeated the experiment, including treatment with the Cx36 blocker, Mef, and using SARM1-KO DRG neurons as a control. Degeneration process was monitored and imaged, and only the 24-h images were posted. The results showed that treatment with Mef dramatically impaired the protective effect, leading to similar levels of degeneration in the injured axons to the double-cut group (Fig. 4c, d, L5 vs L2/L7), while uninjured axons remained intact (Fig. 4c, d, R5 vs R2/R4), highlighting the contribution of gap junctions-mediated communication in protection by adjacent healthy axons. The SARM1-KO DRG neurons maintained their integrity in both axotomy and Mef treatment (Fig. 4c, d, L3 and L6), confirming that SARM1 mediates the injury-induced axon degeneration.

We thus further validate the role of Cx36 using a knockdown approach, employing the same AAV virus as used in Fig. 3f. DRG neurons from wildtype mice were seeded as two separate islands and infected with the AAV virus. Once the axons made contacts, axotomy experiments were conducted similar to those described in Fig. 4a. Successful virus infection was confirmed by the presence of mScarlet fluorescence in the axons (Supplementary Fig. S3c). The fragmentation of the injured axons with Cx36 knocked down was significantly accelerated compared to axons infected with the virus expressing scramble shRNA (Supplementary Fig. S3c, Fig. 4e, L5 vs L2). The fragmentation was mediated by SARM1, as demonstrated by the intact morphology of SARM1-KO axons (Supplementary Fig. S3c, Fig. 4e, L6 vs L3). The knockdown experiments faithfully reproduced the results of treatment with the Cx36 blocker, Mef, strongly suggesting that adjacent healthy axons can protect cut axons from degeneration through intercellular communication via Cx36. To rule out the possibility that Cx36 knockdown itself affects injury-induced axon degeneration, we conducted a time-course study on a single island of neurons. The results showed that there was no significant influence (Supplementary Fig. S3d, e).

The findings highlighted the role of intercellular nucleotide communication in modulating SARM1 activity, thereby enabling healthy neurons to protect adjacent injured neurons. This novel mechanism of SARM1 regulation and neuroprotection is summarized in Fig. 4f.

### Cx36 protects neurons from neuroinflammation, SARM1 activation and axon degeneration

It is well documented that GJ are abundantly expressed in neurons, especially in cell bodies and dendritic regions [40, 41]. Intercellular communication via metabolite transfer as described above most likely occurs in vivo as well, exerting similar protective effect against AxD. To evaluate the protective role of GJIC in mouse brain, we proceeded to knockdown Cx36 and assessed the effects. This was accomplished by using the PHP.eB AAV vector, carrying a Cx36-specific shRNA sequence or a scramble sequence as a comparative control. This vector, a derivative of AAV9, exhibits enhanced CNS tropism, facilitating gene delivery in vivo across the blood-brain barrier [42]. The virus was administrated via the tail vein infusion into both wildtype and SARM1-KO mice. The reason we chose the viral shRNA technique to knockdown Cx36 in mouse brain instead of direct knockout of the gene was to avoid the well documented compensatory mechanisms likely developed in knockout mice. This was of particular concern since there are many alternate forms of Cx in addition to Cx36.

**Fig. 5:**
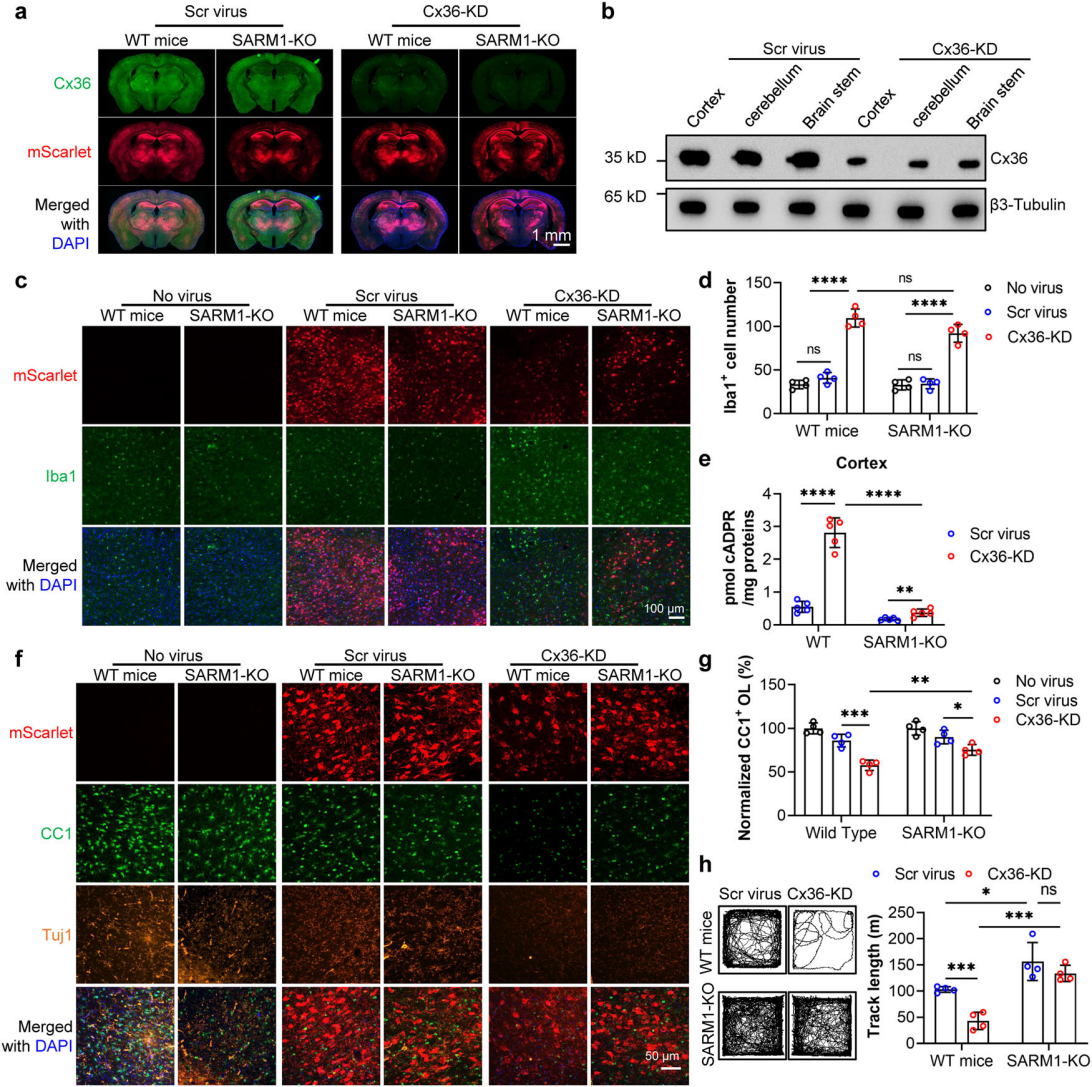
Cx36 inhibits neurotropic virus-induced neuroinflammation, SARM1 activation and behavioral defects in mice. **a** Wildtype mice were infused with AAV-PHP.eB virus carrying scramble or Cx36-specific shRNA via tail vain. After eighteen days, the mice were sacrificed, and perfused with 4% PFA for fixation, and brain sections were immunostained for Cx36. Fluorescent images were captured under a Slide Scanner. Scale bar: 1 mm. **b** Tissues from cortex, cerebellum and brainstem were collected, lysed, and subjected to Western blot analysis to measure Cx36 expression. **c** Frozen brain sections were immunostained for Iba1, a microglial activation marker. Scale bar: 100 μm. **d** The number of Iba1-positive cells in **c** was quantified using ImageJ. **e** Cortical tissue samples were homogenized with 0.6 M perchloric acid, and the concentration of cADPR was measured using a cycling assay. **f** The same brain sections were immunostained with anti-CC1 and anti-Tuj1 antibodies to visualize oligodendrocytes and neurons, respectively. Images were captured using a confocal microscope. Scale bar: 50 μm. **g** The number of CC1-positive cells in **f** was quantified using ImageJ. **h** Wildtype and SARM1-KO mice infused with the virus underwent the open field test, during which their track length was recorded over a 30-minute running session. All experiments were repeated at least three times, and data are presented as means ± SDs (n = 3). Statistical significance was determined using Student’s *t* test in **d**, **e**, **g** and **h**, following one-way ANOVA for comparisons within groups and two-way ANOVA for comparisons between groups (ns no significance; *P < 0.05; **P < 0.01; ***P < 0.001; ****P < 0.0001).

Three weeks post-infusion, brain tissues from mice were analyzed by immunostaining or Western blot. Infection (the mScarlet fluorescence) was predominantly observed in the brain (Fig. 5a and Supplementary Fig. S4b, red) and peripheral nerves (Supplementary Fig. S5a, red), but much less so in other organs (Supplementary Fig. S5b, red), as expected. Immunostaining and Western blots confirmed the presence of Cx36 throughout the brains of the control mice, including in the cortex, cerebellum and brainstem (Fig. 5b), as well as the sciatic nerves (Supplementary Fig. S5a, green), but was markedly diminished in Cx36-KD mice (Fig. 5a and Supplementary Fig. S4b, green). Cx36 staining did not yield conspicuous signals in the lung and pancreas (Supplementary Fig. S5b, green).

The most prominent abnormality observed in Cx36-KD mice was neuroinflammation, as indicated by immunostaining of the microglial activation marker, Iba1 [43], which level was markedly increased as compared with that in the control mice infused with virus containing a scramble shRNA (Fig. 5c, green). The results indicated that the neuroinflammation was specific for Cx36 knockdown and not just due to viral infection. Inflammation was apparently confined to the nervous system, as indicated by the unchanged levels of blood inflammation markers such as IL-6 and IFN-γ (Supplementary Fig. S5c, d). The results show that GJIC via Cx36 plays an important role in protecting the brain from neuroinflammation.

The Cx36-KD-induced neuroinflammation was observed in both wildtype and SARM1-KO mice, as indicated by the statistically similar levels of Iba1 (Fig. 5d). However, the subsequent inflammation-induced cell loss was critically dependent on SARM1, which activity was measured by the levels of cellular cADPR, its enzymatic product [21, 44]. Cx36-KD virus not only induced neuroinflammation but also activated SARM1. In the cortex, cerebellum, and brainstem of the Cx36-KD mice, the cellular levels of cADPR were substantially augmented. That the augmentation was mediated by activation of SARM1 since no such change was seen in SARM1-KO mice infused with the Cx36-KD virus (Fig. 5e and Supplementary Fig. S5e, f).

Cx36-KD virus induced activation of SARM1 should lead to AxD. This was indeed the case. We monitored the cell number of premyelinating and myelinating oligodendrocytes by their marker, CC1 and the marker Tuj1 for neurons the brain slices by immunostaining. The results revealed that the Cx36-KD virus significantly diminished the CC1 signals in wildtype mice but not in SARM1-KO mice (Fig. 5f, green; Fig. 5g). The Tuj1 signals exhibited a similar trend, albeit less pronounced (Fig. 5f, orange). Correspondingly, behavioral tests, the open field and rotarod, showed demised performance of Cx36-KD virus-infused mice compared to those administered with the Scramble virus. SARM1-KO mice infused with Cx36-KD virus, however, were rescued and exhibited essentially normal performance (Fig. 5h and Supplementary Fig. S5g).

## Discussion

Recent progress in understanding of the enzymatic and structural aspects of SARM1 has revealed a possible scenario during axonal degeneration. It is now well documented that SARM1 is an enzyme that utilizes NAD as a substrate, catalyzing multiple reactions, including hydrolyzing it to ADP-ribose, cyclizing it to a calcium messenger, cADPR [45], as well as exchanging the nicotinamide base to produce another calcium messenger, NAADP [19, 21, 46]. SARM1 is auto-regulated and is activated by NMN or its synthetic analog, CZ-48 [21]. In addition to being a substrate, NAD also binds to an allosteric site in the regulatory ARM domain, locking it in an enzymatically inactive form [22]. NMN competes for the same site, leading to the release of the ARM domain from the catalytic TIR domain, and resulting instead, in activation of the enzymatic activities [24–26]. After injury, axonal NAD decreases and NMN increases possibly due to the loss of NMNAT2 [27]. SARM1 is activated as a result, further depleting axonal NAD and leading eventually to AxD.

This scenario focuses only on intracellular events. Both NAD and NMN are small metabolites and should be permeant through GJs, which is well established to allow the flux of molecules between cells as big as 1.5 kDa in molecular weight [47], including metabolites (e.g. ATP), nutrients (e.g. glucose), and second messengers (e.g. IP_3_ and Ca^2+^) [48]. In this study, we initiated the first investigation on the possible role of intercellular transfer of NAD and metabolites in regulating SARM1 and AxD. The evidence is summarized here. We documented and visualized the transfer using the fluorescent analog of NAD, PAD11, endogenously produced by SARM1 via the base-exchange reaction [35, 36]. The transfer was observed not only between somatic HEK-293 cells but also between DRG neurons, indicating it as a general cellular process. Using chemical inhibitor, gene knockout and shRNA knockdown, we showed that, in HEK-293 cells, it was mainly mediated by Cx43, but by Cx36 in neurons, both forms are the dominantly expressed type in the respective cells. The transfer is cell type selective as it is not observed between heterologous cells, such as between HEK-293 and SH-SY5Y expressing different forms of connexins.

We further demonstrated the significant role of intercellular transfer of nucleotides in regulating SARM1 and protecting against AxD. High NAD level resulting from overexpressing NMNATs not only inhibited SARM1 in the overexpressing cells but also affected neighboring wildtype cells in direct contact. This intercellular inhibition of SARM1 has crucial functional consequences in neurons, as healthy DRG axons with normal NAD production could protect injured axons in contact from degeneration. This protective mechanism involves the transfer of regulatory NAD/NMN from the healthy axons, blocking SARM1 activation in injured ones. The physiological relevance of the above results was implied by the presence of Cx36 in sciatic nerve tissue (Supplementary Fig. S5a) and the reported chemical coupling of sensory neurons [40].

Herein, we employed a novel in vitro dual-island neuronal culture technique to investigate inter-neuronal communication. By strictly controlling variables, including neuronal island placement, contact zones, and imaging areas, we were able to distinguish axons and their branches from each side. The reliability of this method was confirmed through inhibitor and gene knockdown experiments (Figs. 3 and 4). However, we acknowledge the limitation that axons from the right island may still enter the left imaging field. To further disseminate this methodology, we have published a comprehensive protocol, detailing the experimental procedures and specific parameters in the protocols.io repository [49].

Furthermore, we explored the protective function of Cx36 in a mouse model. It has been reported that the neurotropic viruses, such as West Nile virus [50] or Zika virus [51], trigger SARM1-mediated axon degeneration. In our experiment, infusion of the neurotropic AAV-PHP.eB virus carrying the Cx36-specific shRNA sequence induced significant neuroinflammation, which in turn activated SARM1 and led to subsequent axon degeneration. In the presence of Cx36, as in the scramble virus-infused mice, the neuroinflammation, together with the subsequent SARM1 activation and axon degeneration, was prohibited. The data suggested the in vivo neuroprotective effect of Cx36-mediated GJIC, which may indirectly inhibit SARM1 activation by reducing neuroinflammation. The GJIC might occur not only between neurons but also between neurons and microglia, which expresses low levels of Cx36 [52].

Overall, our finding highlights the importance of investigating tissue as a whole in neuronal injury and diseases, providing new possibilities for therapeutic interventions targeting gap junctions and intercellular metabolic exchange.

## Materials and methods

Seen in Supplementary Material, including reagents, animal and cell culture, constructs, transient transfection and construction of stable cell lines, virus preparation and infection, nucleotides extraction and cycling assay, western blots, immunostaining and imaging, visualization of PAD11 communication, axotomy on DGR axons, open field test, rotarod test, immunohistochemistry for fixing and sectioning frozen tissues, and data analysis.

## Supplementary information

Supplementary materials

Original files of Western blots

## Data Availability

Supplementary information is available at Cell Death and Disease’s website.
