# Supplementary material for: Gap junction intercellular communications regulates activation of SARM1 and protects against axonal degeneration
Source: Cell Death Dis. 2025 Jan 14;16(1):13. doi: 10.1038/s41419-025-07342-4 (PMC11733139; doi:10.1038/s41419-025-07342-4)
Supplement: Supplementary file 1 — Supplementary materials [file 41419_2025_7342_MOESM1_ESM.docx]

**Supplementary materials**

1. **Materials and methods**

**Reagents**

Lipofectamine 2000 (11668019), Dulbecco's Modified Eagle Medium (DMEM, 12800017), trypsin (25200072), penicillin/streptomycin solution (15070063), laminin (23017015), Neurobasal media (21103049), B-27 (17504001), GlutaMAX (35050061), Rabbit anti-CX36 (QG219843), and Alexa Fluor-conjugated secondary antibody were obtained from Thermo Fisher Scientific (Massachusetts，USA). Neuron growth factor (NGF, 50385-MNAC) was purchased from SinoBiological (Beijing, China). Fetal bovine serum (FBS, P04-96950) was purchased from PAN Biotech (Aidenbach, Germany). Mouse monoclonal anti-NMNAT1 (sc-271557), anti-Cx45 (sc-374354), anti-beta3-tubulin (sc-51670) and anti-HSP90 (sc-69703) were purchased from Santa Cruz Biotechnology (Texas, USA). Rabbit anti-Cx43 (26980-1-AP), anti-Cx46 (19756-1-AP) and anti-beta-tubulin (10068-1-AP) were obtained from Proteintech (Chicago, USA). Rabbit anti-SARM1 (13022) was purchased from Cell Signaling Technology (Massachusetts，USA). Rabbit anti-Iba1 (019-19741) was purchased from Fujifilm (Tokyo, Japan). Rabbit anti-CC1 (OB-PRB070-02) was purchased from Oasis (Columbus, USA). Mouse anti-Tuj1 (801202) was purchased from Biolegend (California, USA). NADase was prepared in the lab from *Neurospora crassa*. Diaphorase (D5540), anti-Flag (F7425), NAD (NAD100-RO), nicotinamide (N3376), digitonin (D141), poly-L-lysine (P1274), poly-D-Lysine (P0899), 4′,6-diamidino-2- phenylindole dihydrochloride (DAPI, 32670), 5-fluoro-2’-deoxyuridine (F0503) and uridine (U3003) were obtained from Sigma-Aldrich (Massachusetts，USA). Mefloquine (S4420) was purchased from Selleck (Shanghai, China). ELISA kits for IL-6 (abs520004) and IFN-γ (abs520004) were obtained from Absin (Shanghai, China). Other chemicals were purchased from Sangon Biotech (Shanghai, China).

**Animal and cell culture**

C57/BL6J mice were obtained from Guangdong Medical Laboratory Animal Center (Guangzhou, China) and SARM1-KO mice were kindly provided by Prof. Wenwen Zeng in Tsinghua University (#018069, The Jackson Laboratory, Maine, USA). The mice were handled in accordance with the guidelines of the Institutional Animal Care and Use Committee (IACUC) at Peking University Shenzhen Graduate School (Protocol #AP0015001).

Dorsal root ganglion (DRG) neurons were isolated from embryonic mice at E12.5-14.5 and cultured following previously described protocols^1^. Briefly, cells were seeded on poly-D-Lysine and laminin-coated plates and maintained in Neurobasal media in a standard humidified tissue culture incubator at 37 °C with 5% CO_2_. The culture medium was supplemented with 2% B-27, 2 mM GlutaMAX, 50 ng/mL NGF, 1 μM 5-fluoro-2’-deoxyuridine, and 1 μM uridine. Every two days, 50% of the culture medium was replaced with fresh one.

NMNAT1-KO HEK-293T^2^, SARM1-OE HEK293 cells^2^, wildtype HEK-293T, HEK-293, and SH-SY5Y cells were cultured in DMEM supplemented with 10% FBS and 1% penicillin/streptomycin solution. The cells were maintained in a standard humidified tissue culture incubator at 37 °C with 5% CO_2_.

**Constructs**

The expression vectors, pCMV-Flag-NMNAT1, -NMNAT2 and -NMNAT3, were generously provided by Prof. Mathias Ziegler from University of Bergen. To generate NMNAT1 mutants (K57G, W169G, N219G, I221G and S16G), mutagenesis was performed on the parent plasmid, pCMV-Flag-NMNAT1, with the Fast Mutagenesis System Kit (Transgene, Beijing, China). For the Cx43-rescueing plasmid, the DNA sequence adjacent to the PAM region (5’-ATTTTCCGAATCCTGCTGC-3’) was replaced with 5’-ATCTTTAGAATATTATTAT-3’ to preserve the encoded peptide sequence while avoiding editing by CRISPR/cas9.

**Transient transfection and construction of stable cell lines**

For transient transfection of cells, Lipofectamine 2000 was used following the manufacturer’s instructions.

To generate Cx43-KO, Cx45-KO, and Cx46-KO cell lines, specific sgRNAs (Table 1) were cloned into an all-in-one CRISPR plasmid, pSpCas9(BB)-2A-Puro (#48139, Addgene, Massachusetts，USA). HEK-293T cells were transfected with the plasmids and single colonies were generated through serial dilution. Gene knockout was confirmed by Western blot using antibodies against connexins, as well as genomic DNA sequencing.

| Table 1. Primers for knocking out connexins and validation | |
| --- | --- |
| Cx43-gRNA-F | 5’ CACCGCATTTTCCGAATCCTGCTGC |
| Cx43-gRNA-R | 5’ AAACGCAGCAGGATTCGGAAAATGC |
| Cx43-F (gDNA sequencing) | 5’ ACTTGTTAGATCTTTTCTTCGTTGGCA |
| Cx43-R (gDNA sequencing) | 5’ TTTCACCTTACCATGCTCTTCAATACC |
| Cx45-gRNA-F | 5’ CACCG ACAACCATTCCACATTTGTG |
| Cx45-gRNA-R | 5’ AAAC CACAAATGTGGAATGGTTGTC |
| Cx45-F (gDNA sequencing) | 5’ AGTTGGAGCTTCCTGACTCG |
| Cx45-R (gDNA sequencing) | 5’ CCAGCAACTGCAGCACATAG |
| Cx46-gRNA-F | 5’ CACCG GCTGTTCATCTTCCGCATCT |
| Cx46-gRNA-R | 5’ AAACAGATGCGGAAGATGAACAGCC |
| Cx46-F (gDNA sequencing) | 5’ CGACTGGAGCTTTCTGGGAA |
| Cx46-R (gDNA sequencing) | 5’ GAGGGATTGTCCTGCGGTG |

HEK-293 cells stably expressing EGFP, mCherry or SARM1, and SH-SY5Y stably overexpressing Cx43 were also generated through lentivirus infection and puromycin selection, following previously described methods ^1^.

**Virus preparation and infection**

To decrease Cx36 expression in vivo, we used the pAAV-PHP.eB-U6-shRNA (Gjd2)-CMV-mScarlet-WPRE vector (Cx36-KD) from Obio Technology, while the pAAV-PHP.eB-U6-shRNA (NC2)-CMV-mScarlet-WPRE vector was used as a control (Scramble). The sequence of the shRNA targeting Cx36 is ACCGACCGTTACCCCTGCATCAATTCAAGAGATTGATGCAGGGGTAACGGTTTTTTTG, and the sequence of the scrambled shRNA is ACCGCCTAAGGTTAAGTCGCCCTCGCTCGAGCGAGGGCGACTTAACCTTAGGTTTTTTG. The AAV virus was purified by ultracentrifugation and diluted in 0.9% saline to a final volume of 200 μL with a final titer of 2 × 10^11^ viral genome and injected into each mouse intravenously. Behavioral, imaging and characterization experiments were performed at least 18 days after injection. To infect DRG neurons *in vitro*, virus with the same multiplicity of infection (MOI) was added to DRG islands during seeding and further experiments were performed on Div6-8.

**Nucleotides extraction and cycling assay**

Nucleotides, including NAD, cADPR, and NMN, were extracted and quantified using previously described methods^2^. For cell samples, lysis was performed by vortexing in 0.6 M perchloric acid, while brain tissue samples were homogenized using a Tissuelyser-24 homogenizer (Jingxin, Shanghai, China) in 0.6 M perchloric acid. Following centrifugation, the supernatant was used for quantification of nucleotides and pellets for proteins. NAD levels were measured using a cycling assay^3^, while cADPR and NMN were quantified by cycling assay following the conversion to NAD by recombinant ADP-ribosyl cyclase^3^ and NMNAT1^4^, respectively. The protein pellets from centrifugation were re-dissolved in 1 M NaOH and quantified using the Bradford assay (#5000201, Bio-rad, California, USA). Results were expressed as picomole of cADPR, NMN or NAD per milligram of total proteins.

**Western blots**

Cells were lysed with RIPA buffer (50 mM Tris-HCl, 150 mM NaCl, 1 mM EDTA and 0.05% Triton X-100, pH 7.4) with Roche (Basel, Switzerland) cOmplete™ Protease Inhibitor Cocktail. The lysates were denatured and loaded onto 12% SDS-PAGE gels and the proteins were transferred to PVDF membranes. The membranes were then blocked with 5% milk and incubated with primary antibodies against Flag tag, NMNATs, connexins or SARM1, with Tubulin and HSP90 used as internal controls. After incubation with HRP-conjugated second antibodies, the signals were visualized using ECL detection system (Abvansta, California, USA) and captured with a Chemidoc MP system (Bio-rad). The band intensities were quantified using ImageLab software (Bio-Rad).

**Immunostaining and imaging**

HEK-293 cells expressing NMNATs or SARM1 were seeded on poly-L-lysine-coated coverslips. After 48 hours of transfection or 8 hours of co-culture, the cells were fixed in 4 % paraformaldehyde (PFA) for 15 min and permeabilized with 0.1% Triton X-100 in PBS for 5 min. Following blocking with 10 mg/ml BSA for 1 h, the cells were incubated with anti-Flag or anti-SARM1 at room temperature for 1.5 h. Subsequently, the cells were incubated with Alexa Fluor dye-conjugated secondary antibodies. DAPI staining was performed for 5 min to visualize the nuclei. Finally, the coverslips were mounted and the cells were imaged using confocal microscopy (Nikon, Tokyo, Japan).

**Visualization of PAD11 communication**

HEK-293 or SH-SY5Y cells stably expressing mCherry or SARM1 were cultured separately or mixed in a 1:1 ratio in 8-well Chambered Coverglass (#155411, Thermo Fisher). The cells were treated with 100 μM CZ-48 and 12.5 μM PC11 for 8 hours. Fluorescence signals were analyzed using a confocal microscope (Nikon) or a flow cytometer (CytoFlex, California, USA) with specific parameters for PAD11 (445 nm excitation and 595 nm emission) or mCherry (561 nm excitation and 610 nm emission).

DRG neurons were seeded in the corners of an 8-well Chambered Coverglass to create two separated islands. After allowing the axons project and make contacts, the cultures were then treated with 25 μM PC11 with or without 200 μM CZ-48 for 16 h. Images were captured using a confocal microscope (Nikon) with the appropriate settings for PAD11 or mCherry fluorescence as mentioned earlier. The regions of interest for imaging were selected near the somatic region, beyond the zone where axons form contacts, with axon angles ranging between 30 and 60 degrees. The fluorescence of PAD11 was normalized to the area of the axons observed in the bright field. For the connexin blockage experiment, the neurons were pre-treated with 5 μM Mefloquine for 2 h prior the addition of 200 μM CZ-48 and 25 μM PC11. In the Cx36-knockdown experiment, AAV-PHP.eB encoding Cx36-specific shRNA was added during the seeding of DRG islands. A full protocol has been deposited in the protocols.io repository^5^.

**Axotomy on DGR axons**

DRG islands were seeded in a 24-well plate and allowed to establish axonal projections and contacts over several days. Axotomy was performed on the left and/or right DRG axons using a knife, and images were captured using an inverted microscope (Nikon). The degeneration index, representing the ratio of the degenerate area to the total area, was calculated using ImageJ as previously described ^1^. For the inhibition or knockdown of Cx36, pretreatments were carried out following the same protocol as described in the PAD11 imaging experiments mentioned earlier. The Degeneration Index (DI) was calculated following the utilization of the Analyze Particles tool in ImageJ to detect neurite fragments of cultured cells.

**Open field test**

The open field test was conducted in an arena measuring 40 cm x 40 cm x 50 cm, with opaque white Plexiglas on all sides and the bottom. Subjects were transferred from their housing room to the testing room and allowed a minimum of 10 minutes to acclimate before testing. The test sessions were recorded using digital video cameras and subsequently analyzed using Viewer software. Prior to each testing session, the arena underwent cleaning with 75% ethanol and was left to dry thoroughly. Testing commenced by placing the subject in the center of the arena. After a 30-minute period, the subject was returned to its home cage.

**Rotarod test**

The rotarod test were conducted using the Ugo Basile RotaRod (Gemonio, Italy) machine. Subjects were situated in the testing room for a minimum of 1 hour before testing to mitigate stress effects on behavior. During days 1 and 2, the apparatus was programmed to accelerate from 4 to 40 rpm over a 300-second interval. On days 3 and 4, the apparatus was programmed to accelerate from 8 to 80 rpm over the same duration. Subjects from the same cage were allocated to separate lanes on the rod, initially rotating at 4 rpm or 8 rpm. The trial commenced upon acceleration initiation and ceased upon animal dismount. In instances where the animal clung to the rod and completed full passive rotation for 2 cycles, the timer was halted, and the animal was reintroduced to its home cage. This procedure was repeated for three trials, with intervals of 1 hour between each trial per day. Speed at fall was documented for subsequent analysis.

**Immunohistochemistry for fixing and sectioning frozen tissues**

To preserve tissue morphology and antigenicity, tissues were fixed by vascular perfusion with more than 10 mL of 4% PFA Fixative Solution. Brain and tissue samples were then collected and immersed in a 30% sucrose solution for dehydration, with the process lasting at least 2 days. The brain was mounted in OCT embedding compound on dry ice and frozen at -80°C. Tissue sections measuring 30 µm thick were sliced from the brain samples using a cryostat set at -20°C. These sections were then thawed in PBS and mounted onto gelatin-coated histological slides. After mounting, the sections were treated with blocking buffer (5% BSA, 0.3% Triton X-100) for 30 minutes. The primary antibody, prepared in blocking buffer, was incubated with the sections in an immunohistochemical wet box at 4°C overnight. Following incubation, the sections were rinsed with PBS and then incubated with a fluorescent secondary antibody for 2 hours. After another rinse with PBS, DAPI fluoromount-G was applied, and the sections were covered with a coverslip. Images were captured using either a Slide Scanner (Olympus VS200, Tokyo, Japan) or a Confocal microscope (Nikon). Cell numbers or percentages were calculated based on counting performed using ImageJ software.

**Data analysis**

Statistical analyses were conducted as detailed in the relevant sections and figure legends, utilizing GraphPad Prism 8.3.0. Data values were derived from a minimum of three independent experiments, each including three technical replicates per condition. For multi-group comparisons, one-way or two-way analysis of variance (ANOVA) was employed before using unpaired Student's *t*-tests, while unpaired Student's *t*-tests were used for two-group comparisons, as specified in the figure legends. All statistical tests were two-tailed, with a *P*-value threshold of less than 0.05 deemed significant. The significance levels are denoted as follows: ns, not significant; *, *P* < 0.05; **, *P* < 0.01; ***, *P* < 0.001; ****, *P* < 0.0001.

1. **Supplementary figures**


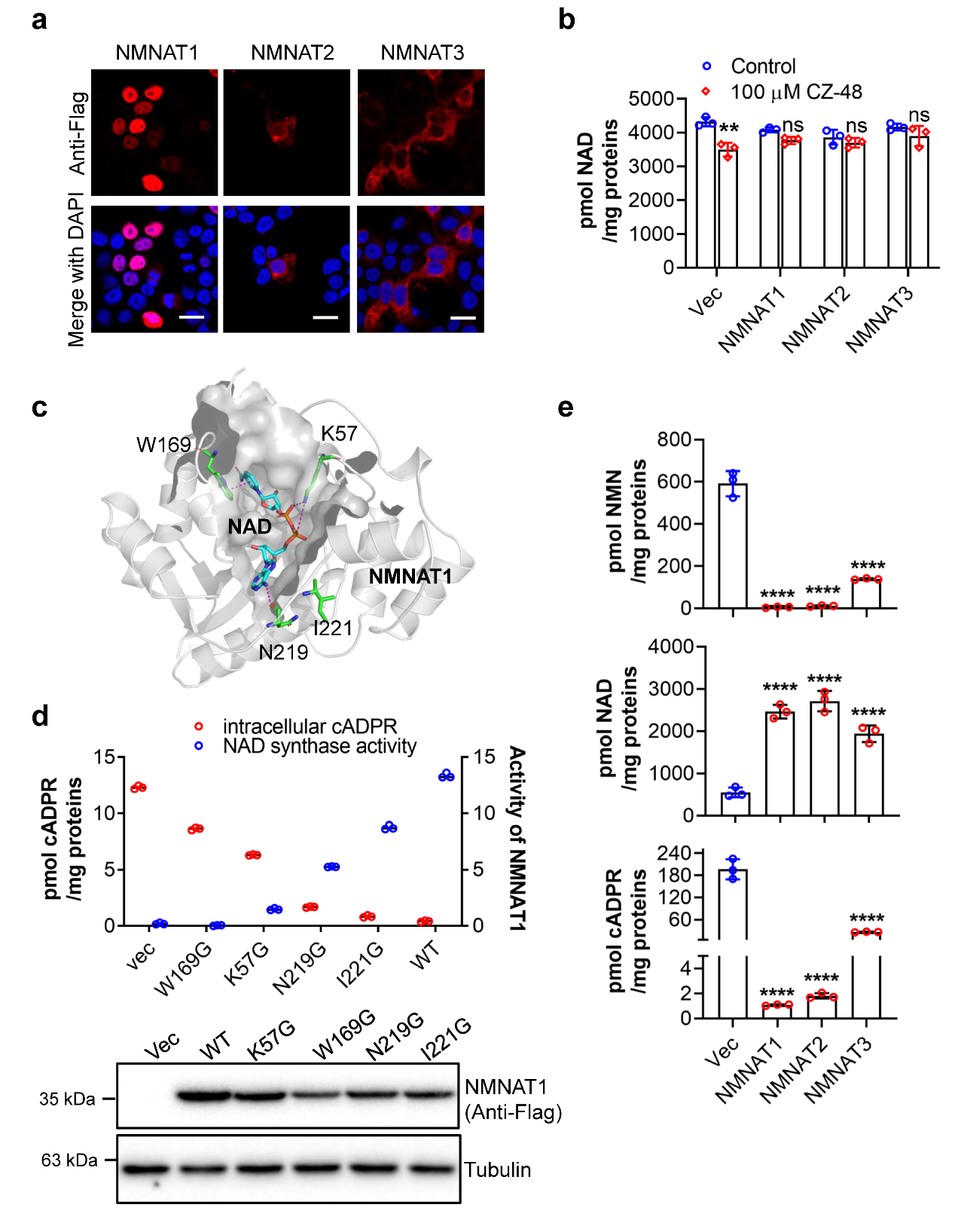


**Fig. S1**. **NMNATs inhibit SARM1’s activation by modulating the cellular NMN/NAD ratio.** **a**, Immunofluorescence staining of cells from (Fig. 1a) using anti-Flag and anti-mouse-Alexa Fluor 555, and DAPI for the nucleus. Scale bar: 20 μm. **b**, Supplementary data for Figure 1a. The transfected HEK-293T cells were treated with 100 μM CZ-48 or PBS and incubated for 24 hours before being lysed. Levels of NAD were measured using a cycling assay. **c**, Crystal structure of the NMNAT1/NAD complex (PDB ID: 1KQN), shown in cartoon mode with the catalytic pocket highlighted in surface mode and key residues depicted in stick mode. **d**, Similar experiments were conducted as in Figure 1A, but HEK-293T cells were transfected with the expression vectors encoding Flag-tagged wildtype and mutant NMNAT1 and additional NAD synthase activities were measured. **e**, NMNAT1 knockout HEK-293T (NMNAT1-KO) cells were transfected with the same vectors used in (a), and the levels of NMN, NAD, and cADPR were measured by the cycling assay after 48 h. All experiments were performed at least three times, and data are presented as mean ± SDs (n ≥ 3). Statistical significance was determined by Student’s *t*-test following one-way ANOVA test in **e**, and Student’s *t*-test in **b** (ns, no significance; **, *P* < 0.01; ****, *P* < 0.0001).


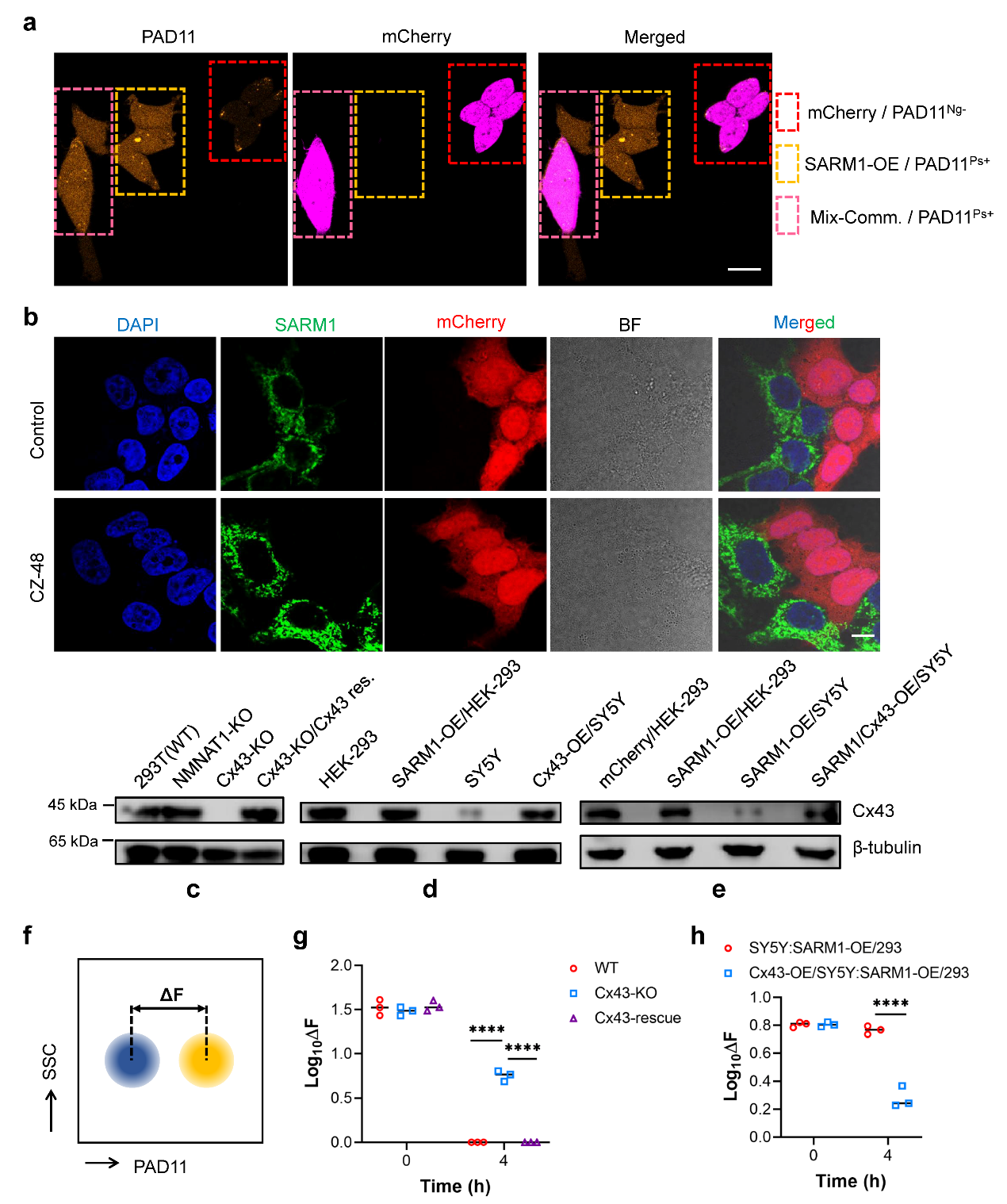


**Fig. S2.** **Supplementary data for Figure 2.** **a**, PAD11 communication between wildtype and SARM1-overexpression HEK-293 cells. The experiments were done as Figure 2b. Three clusters of cells were observed in the same field. Red square: isolated HEK-293 expressing mCherry, without PAD11 signals; orange square: SARM1-overexpression HEK-293 cell carrying the signal of PAD11; Pink square: two contacting cell clusters, with similar intensity of PAD11 signals. Scale bar 10 μm. **b**, The immunostaining of the co-culture of mCherry/HEK-293 and SARM1-OE/HEK-293 cells, following treatment of 100 μM CZ-48 for 8 hours. A monoclonal primary antibody, anti-SARM1, and AlexFluo488-conjugated anti-rabbit secondary antibody were used for staining SARM1, and DAPI is used for nuclear staining. Scale bar: 10 μm. **c-e**, Expression level of Cx43 in the cells used in Fig. 2e-g was measured by Western blot. All experiments were performed at least three times. **f**, The quantification strategy for the flow cytometry results shown in Fig. 2e-f. The value of ΔF was defined as the fluorescence difference between the two population of cells. **g-h**, Quantification of the flow cytometry results in Fig. 2e-f using the strategy shown in f. Statistical significance was determined using Student’s *t*-test following one-way ANOVA test in **g**, and Student’s *t*-test in **h** (****, *P* < 0.0001).


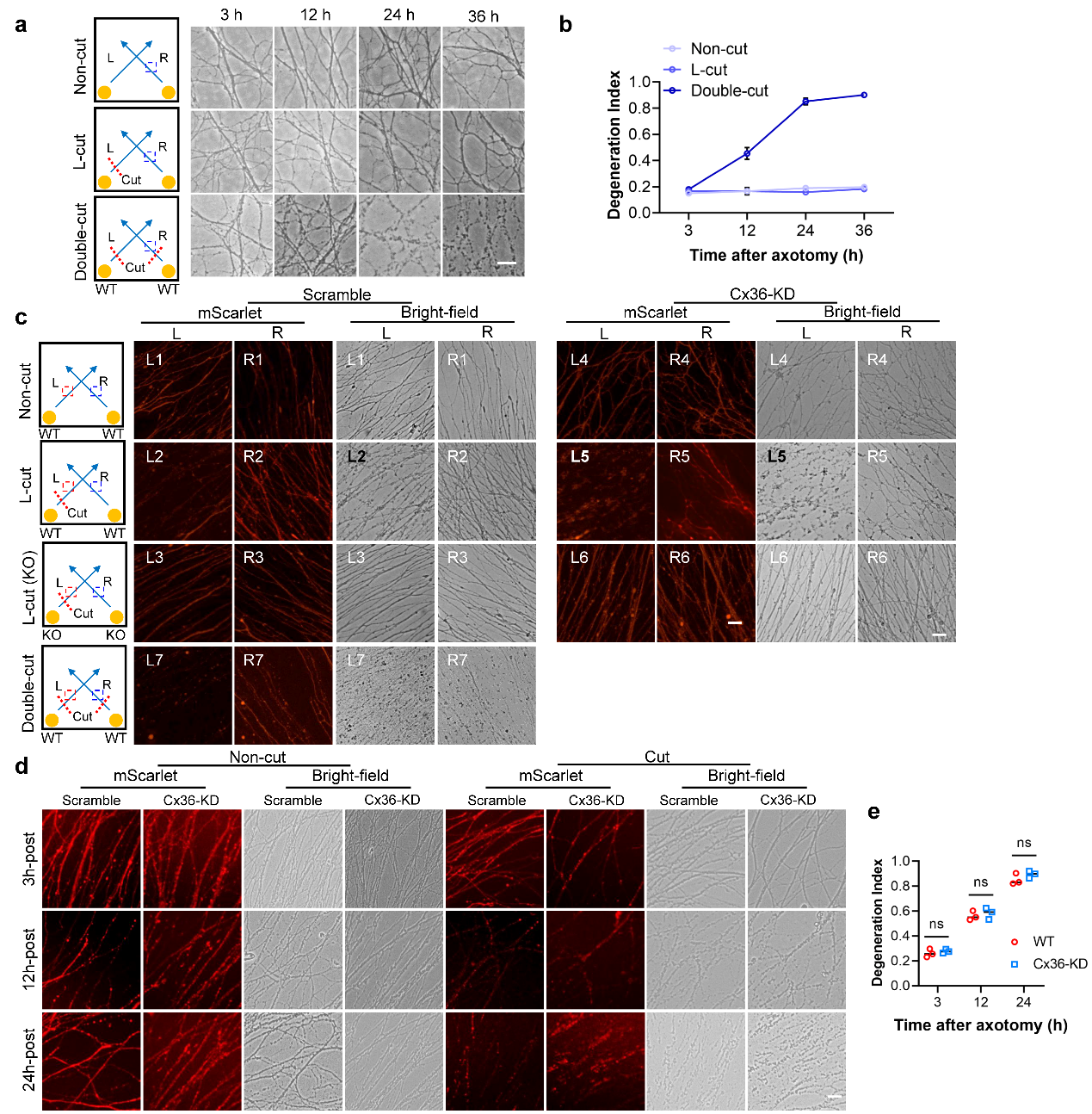


**Fig. S3. Supplementary data for Figure 4**. **a**, In the experiment shown in Fig. 4a, fluorescence and bright-field images were taken from the area (denoted by a blue square) where axons projected from the right island. **b**, Degeneration indices of the axons from (a), were calculated and plotted. **c**, Wildtype DRG neurons were seeded as two islands and infected with AAV-PHP.eB encoding Cx36-specific shRNA, or scramble shRNA, together with an mScarlet expression cassette. After the axons established contacts, axotomy was performed as illustrated. Fluorescence and bright-field microscope images were captured in the squared areas 24 hours post-axotomy. Scale bar: 10 μm. **d**, Wildtype DRG neurons were seeded as a single island and infected with the same virus as (c). After the axons had projected long enough, axotomy was performed. Fluorescence and bright-field microscope images were captured in the distal area of the axotomy at 3, 12, and 24 hours post-axotomy. Scale bar: 10 μm. **e**, Degeneration indices of the axons were quantified by ImageJ and plotted over time. Statistical significance was determined using Student’s *t*-test (ns, no significance).


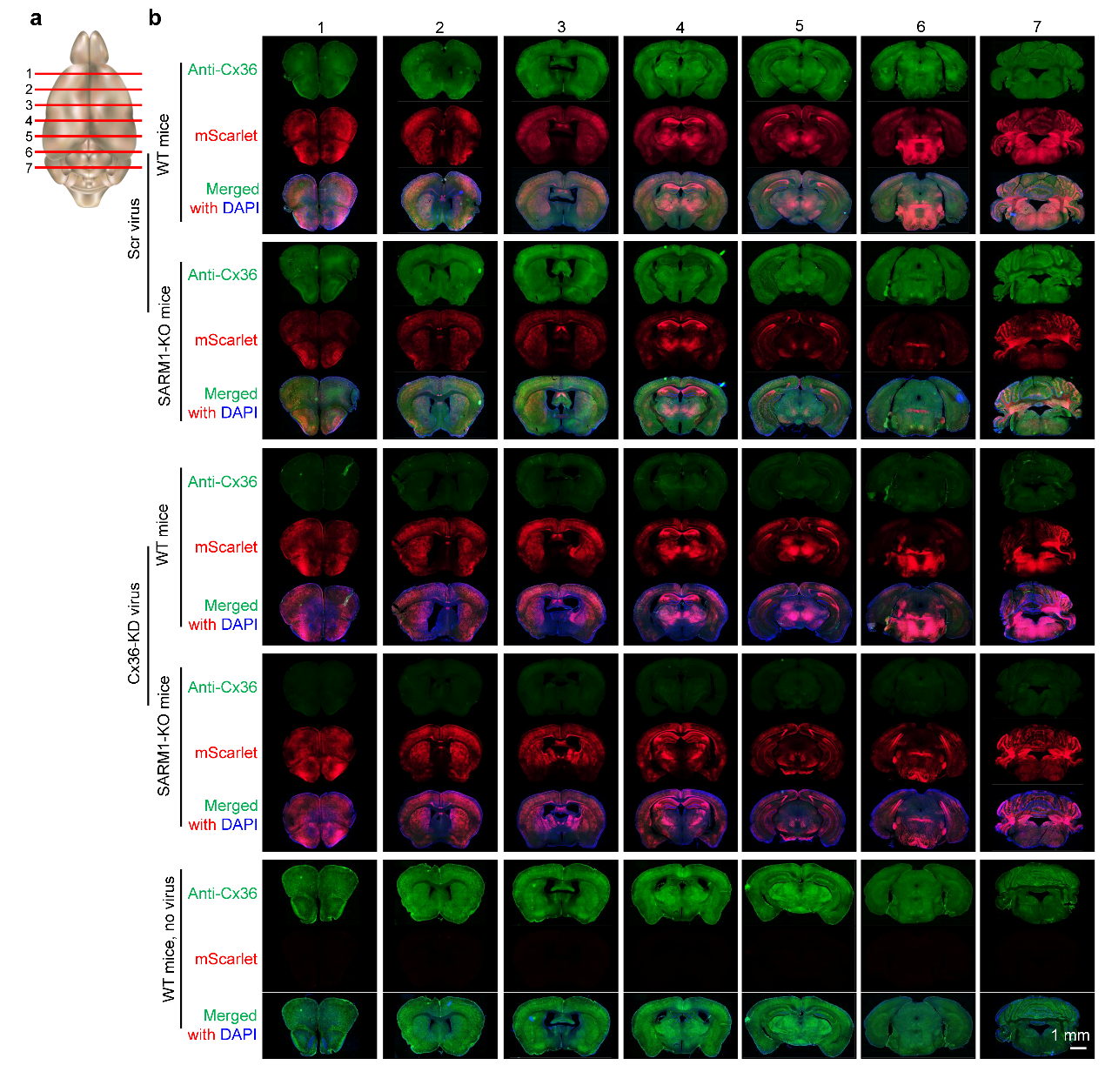


**Fig. S4.** **Supplementary data for Figure 5a**. **a**, Illustration showing the location of different sections. **b**, Same experiments as in Figure 5a. Scale bar: 1 mm.

**
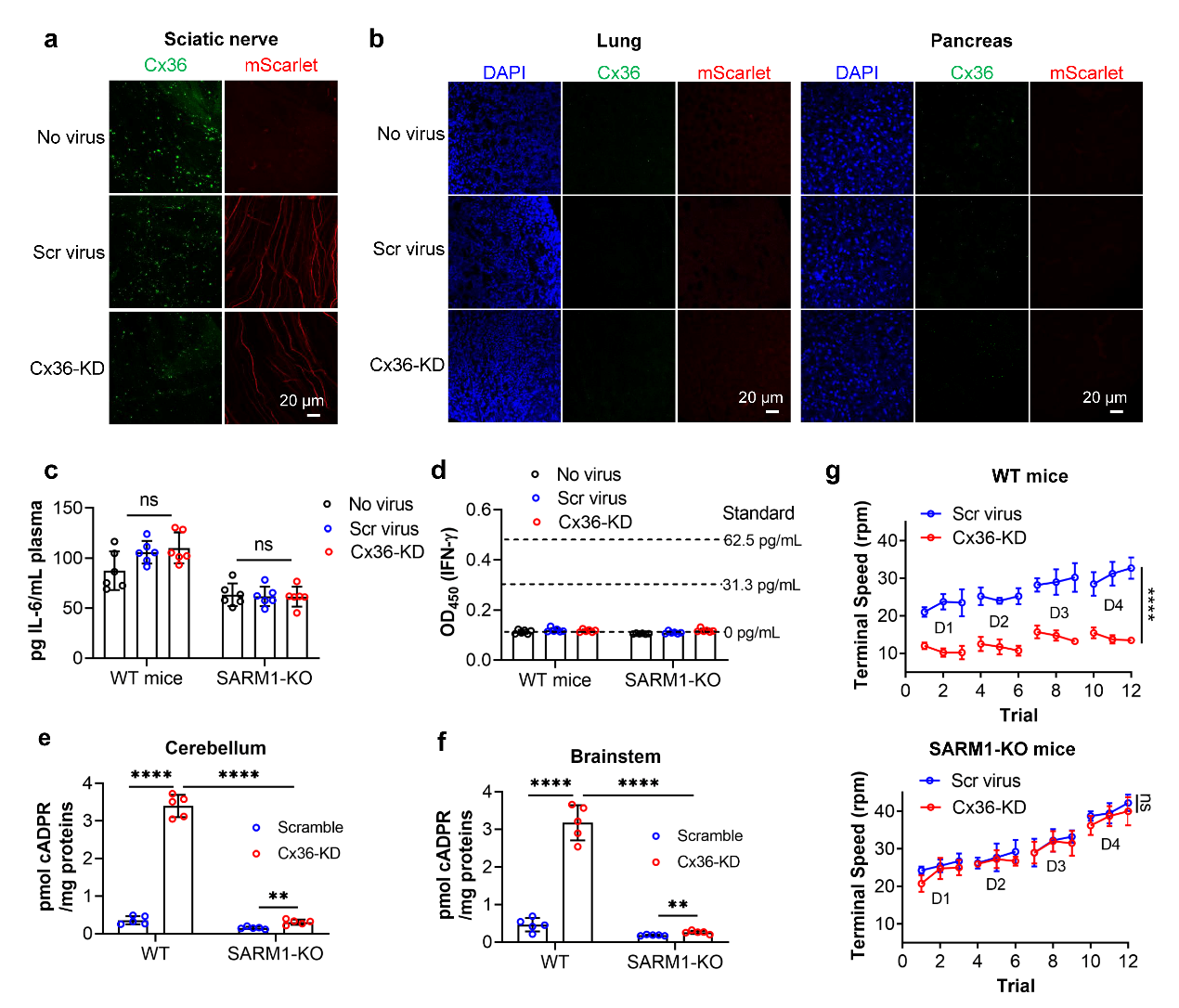
**

**Fig. S5 Supplementary data for Figure 5.** **a-b,** Immunostaining of the fixed sciatic nerve (a) and lung and pancreas (b) with anti-Cx36 antibody. Scale bar: 20 μm. **c-d**, Measurement of IL-6 (c) and IFN-γ (d) levels in mice blood collected from the retroorbital plexus using heparinized capillary tubes, performed using commercial ELISA kits. **e-f**, Measurement of cADPR contents in cerebellum (e) and brainstem (f) tissue samples from the same batch of mice as in Figure 5, using the cycling assay. **g**, Rotarod test performed on the mice in Figure 5H. Mice were trained on the rotarod three times a day for four consecutive days, and the terminal speed at which mice fell from the rod was recorded. Statistical significance was determined using Student’s *t*-test in **c**, **e**, and **f**, following one-way ANOVA for comparisons within groups; Student’s *t*-test following two-way ANOVA to test the significance for comparisons between groups in **e**-**g** (ns, no significance; **, *P* < 0.01; ****, *P* < 0.0001).

1. **Reference for supplementary materials**

1 Li WH, Huang K, Cai Y, Wang QW, Zhu WJ, Hou YN *et al.* Permeant fluorescent probes visualize the activation of SARM1 and uncover an anti-neurodegenerative drug candidate. *eLife* 2021; **10**: e67381.

2 Zhao ZY, Xie XJ, Li WH, Liu J, Chen Z, Zhang B *et al.* A Cell-Permeant Mimetic of NMN Activates SARM1 to Produce Cyclic ADP-Ribose and Induce Non-apoptotic Cell Death. *iScience* 2019; **15**: 452–466.

3 Graeff R, Lee HC. A novel cycling assay for cellular cADP-ribose with nanomolar sensitivity. *Biochem J* 2002; **361**: 379–384.

4 Zamporlini F, Ruggieri S, Mazzola F, Amici A, Orsomando G, Raffaelli N. Novel assay for simultaneous measurement of pyridine mononucleotides synthesizing activities allows dissection of the NAD(+) biosynthetic machinery in mammalian cells. *FEBS J* 2014; **281**: 5104–5119.

5 Zhu WJ, Li WH, Lee HC, Zhao YJ. Protocol for investigating neuronal communication via gap junctions. protocols.io 2024; dx.doi.org/10.17504/protocols.io.eq2ly6jorgx9/v1.
